# Impact of an inpatient nurse-initiated penicillin allergy delabeling questionnaire

**DOI:** 10.1017/ash.2022.55

**Published:** 2022-05-20

**Authors:** Hilary Bediako, Lauren Dutcher, Aditi Rao, Kristen Sigafus, Christina Harker, Keith W. Hamilton, Olajumoke Fadugba

**Affiliations:** 1 University of Pennsylvania Perelman School of Medicine, Philadelphia, Pennsylvania; 2 Division of Infectious Diseases, Department of Medicine, University of Pennsylvania Perelman School of Medicine, Philadelphia, Pennsylvania; 3 Department of Biostatistics, Epidemiology, and Informatics, University of Pennsylvania Perelman School of Medicine, Philadelphia, Pennsylvania; 4 Department of Nursing, Hospital of the University of Pennsylvania, Philadelphia, Pennsylvania; 5 University of Pennsylvania School of Nursing, Philadelphia, Pennsylvania; 6 Division of Pulmonary, Allergy and Critical Care, Department of Medicine, University of Pennsylvania Perelman School of Medicine, Philadelphia, Pennsylvania

## Abstract

Penicillin allergy delabeling is an important component of antimicrobial stewardship and improves patient outcomes. We demonstrated the successful use of a nurse-initiated questionnaire to remove inappropriate penicillin allergy labels in inpatients. Nurses can play a key role in improving antibiotic allergy assessment and more broadly in interprofessional antimicrobial stewardship.

Approximately 10% of patients in the US report an allergy to penicillin, but >90% of these patients tolerate penicillin without an immediate-type hypersensitivity reaction.^1,2^ Beta-lactam antibiotics are often the preferred agent for the optimal treatment of certain infections, and penicillin allergy labels have been associated with adverse outcomes, including higher rates of *Clostridioides difficile* and infections with multidrug-resistant organisms as well as increased length of hospital stay.^2,3^ National and international entities have issued calls to pursue penicillin allergy delabeling as part of antimicrobial stewardship efforts.^4^ Prior studies have focused on physician or pharmacist-initiated penicillin skin tests; none have explored the nurse’s potential role in utilizing allergy history taking to identify patients appropriate for penicillin allergy delabeling in the acute-care setting.^2,5^ Nurses’ presence at the bedside and their early interaction with inpatients uniquely positions them to enhance antimicrobial stewardship efforts.^6,7^ In this study, we assess the impact of utilizing an inpatient nurse-initiated questionnaire to facilitate penicillin allergy delabeling.

## Methods

### Intervention and setting

A nurse-initiated penicillin allergy delabeling questionnaire was implemented in 16 inpatient acute-care units at an academic hospital in Philadelphia, Pennsylvania. Patients at least 18 years of age with a documented penicillin allergy label and receiving care in medical and surgical wards were included. We applied the following exclusion criteria: patients receiving care in intensive care, women’s health, or pediatric units and patients unable to provide medical history or verbal consent, receiving hospice care, or with cystic fibrosis (due to potential complex antibiotic exposure and allergy history). The study was implemented from July 9, 2019, to July 24, 2020.

A questionnaire designed to elicit patients’ allergy histories to assess their eligibility and willingness for penicillin delabeling was created electronically (Supplementary Fig. 1). Clinical nurse specialists (ie, master’s degree–prepared nurses in unit-based leadership roles) administered the questionnaire, which used skip logic to lead them through a series of up to 5 questions. The nurses documented responses in real time. If the responses indicated potential delabeling eligibility, the nurse discussed the patient’s case with an infectious disease or allergy-trained physician. The physicians reviewed the questionnaire responses and made a recommendation regarding whether or not they agreed with the appropriateness of delabeling, which also included label clarification to another β-lactam antibiotic (relabeling) or label clarification to a nonallergic adverse reaction. If physicians agreed and patients were willing to be delabeled (or relabeled), the allergy was removed or clarified with explanatory documentation in the chart. Primary care providers were also notified. Patients who were not willing to be delabeled or who were unable to be safely delabeled by history alone were offered information about follow-up with an allergy provider after discharge.

### Data collection and outcome assessment

Questionnaire responses were collected in a secure electronic database. Following study completion, participating nurses were also surveyed about their experiences administering the questionnaire. The primary outcome was the proportion of patients with a penicillin allergy label who were delabeled. This study was granted exemption by the Institutional Review Board at the University of Pennsylvania.

## Results

In total, 295 patients with penicillin allergy labels were assessed; 64 were excluded, with 4 then participating at a later date. Reasons for exclusion included: hospice (n = 5), cognitive impairment (n = 45), patient refusal (n = 11), and other (n = 3). Of the 235 participating patients, 33 (14.0%) were potentially eligible for penicillin delabeling based on the questionnaire. Reasons for potential delabeling eligibility and ineligibility are listed in Table 1. Of the eligible patients, 6 were appropriate for relabeling rather than delabeling and 7 were eligible for clarification of their nonallergic adverse reaction (Table 1). Of the 33 potentially eligible patients, 17 were amenable to delabeling and 14 declined. Reasons for declining are listed in Table 1. Of the patients who declined, 4 were interested in an outpatient allergy evaluation, 1 was unsure and 7 were not interested. The physician pool agreed with delabeling for 23 of 33 patients and disagreed for 8 patients (Table 1). 15 patients (6.4%) were ultimately delabeled.

**Table 1.** Penicillin Delabeling Questionnaire Outcomes

Penicillin Delabeling Outcomes	Patients, No. (%)
**Reasons for ineligibility for delabeling by questionnaire (N=202)**	
Unable to recall anything about past reaction to penicillin or does not know why it is listed as an allergy	49 (24)
Not known to have subsequently taken penicillin antibiotic without reaction	142 (70)
Failed to fully complete questionnaire	11 (5)
**Reasons for potential eligibility for delabeling (N=33)**	
Never took penicillin and had a reaction—reported family member with allergy	1 (3)
Never took penicillin and had a reaction—reported “other” as reason for allergy label	5 (15)
Reaction was side effect or nonallergic reaction	7 (21)
Subsequently took penicillin antibiotic without reaction	15 (45)
Reaction was not to penicillin but was to another penicillin-type antibiotic (eligible for relabeling)	6 (18)
**Patient agreement to delabeling or relabeling (N=33)**	
Agreed	17 (52)
Declined	14 (42)
No response documented	2 (6)
**Physician specialist agreement to delabeling or relabeling (N=33)**	
Agreed	23 (70)
Disagreed	8 (24)
No response documented	2 (6)
**Reasons for patient refusal of delabeling or relabeling (N=14)** ^**a**^	
I am afraid that I might actually be allergic even though, my answers to the questionnaire indicate that I’m not allergic	3 (21)
I would prefer to be tested (eg, skin test) for penicillin allergy before removing the label	1 (7)
I would never want the allergy label removed no matter what	5 (36)
Other	4 (29)
**Reasons for physician specialist recommendations against delabeling (N=8)**	
Patient not confident in reported allergy history	2 (25)
Already planned for outpatient allergy appointment and skin testing	2 (25)
Eligible for relabeling but physician failed to recommend	1 (13)
Patient already refused	1 (13)
Other	2 (25)

a Categories not mutually exclusive.

**Fig. 1.**
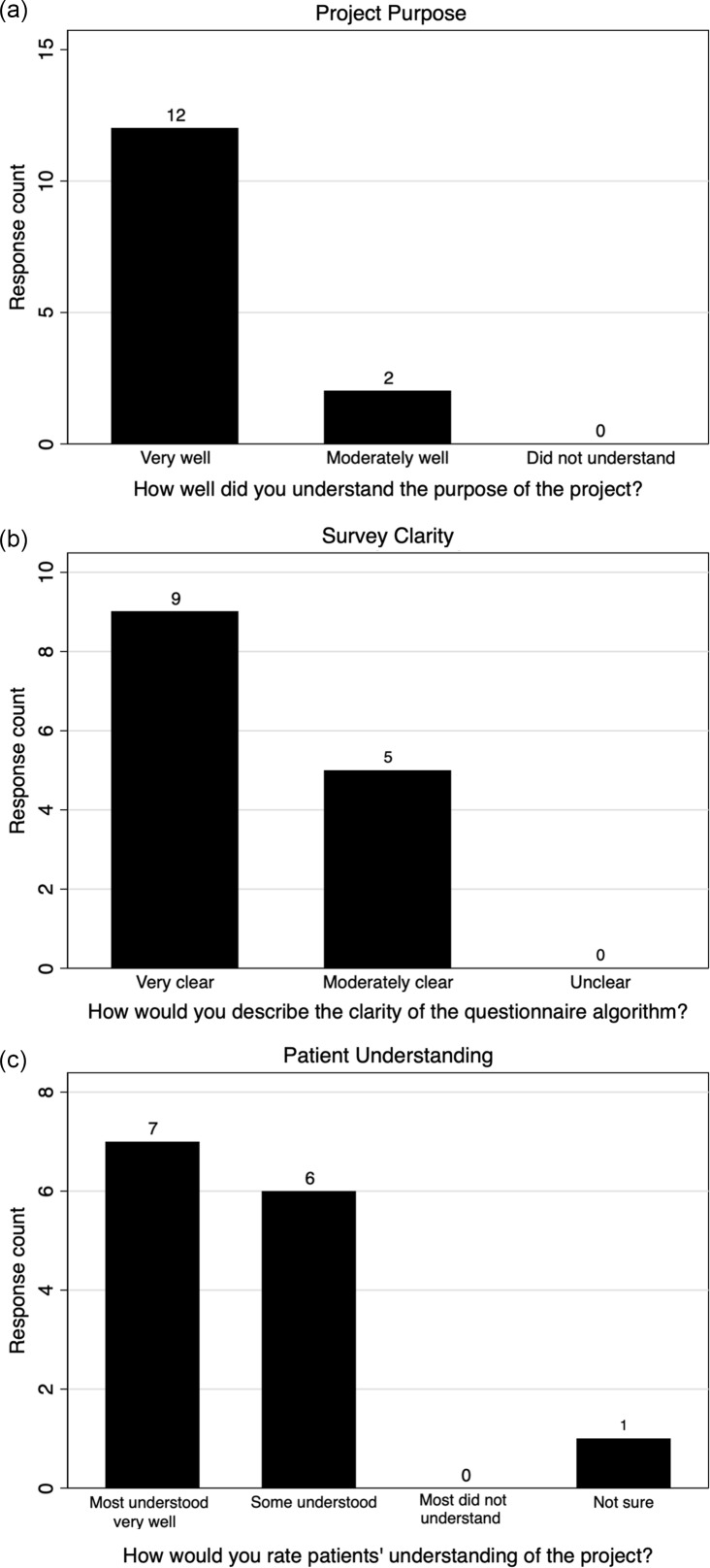
Nurse survey responses regarding experiences administering penicillin allergy questionnaire.

In total, 19 nurses were surveyed about their experiences administering the questionnaire. 14 (73.7%) completed the survey. Of the 14, 11 reported administering it >5 times. 11 nurses reported the questionnaire took “just the right amount of time” to administer, and 3 nurses reported that it took too long. A majority of nurses reported understanding the purpose of the questionnaire very well (12/14), and all noted that the questionnaire algorithm was moderately or very clear (Fig. 1A and B). Most nurses reported that either some or most patients understood the purpose of the survey (Fig. 1C). Nurses also felt that most patients were receptive to receiving information on outpatient penicillin skin testing but provided reasons that patients reported for declining (Supplementary Fig. 2).

## Discussion

We demonstrated that a nurse-initiated penicillin allergy questionnaire can be successfully implemented to delabel or relabel inpatients with penicillin allergy labels based on history alone, without skin testing or oral challenge. Although only a small proportion of patients were delabeled with this questionnaire, these were patients who otherwise were not likely to have had their penicillin allergy labels removed during their hospital admission. This nurse-led and history-driven approach to delabeling also has the additional benefit of not requiring the cost, time, and resources of specialist testing in the appropriate setting. We have shown that nurses can play a key role in allergy assessment, which is critical for designing an interprofessional antimicrobial stewardship approach.^6–8^ Some nurses may be hesitant to override documented drug allergies, but our intervention was performed by nurse leaders and incorporated specialist physician validation, which may be important in implementing a nurse-led intervention.^6,9^ An additional benefit to the nurse-initiated process is that it provided patients with allergy education and an opportunity to undergo further outpatient evaluation, thus having a potentially larger impact beyond bedside evaluation. One limitation of our study was that we did not assess uptake of outpatient allergy evaluation when offered in this context or the long-term durability of delabeling, both of which should be assessed in future interventions.

Interestingly, some patients declined delabeling, with a substantial proportion giving reasons that suggested a lack of trust or interest in allergy delabeling by history. Only some of these patients were amenable to outpatient allergy evaluation and/or skin testing, and others did not ever want their label removed or did not think it was important. Although limited by a small sample size, our findings supplement other studies in highlighting potential patient barriers to penicillin allergy evaluation and delabeling.^9,10^ These findings suggest that effective interventions will likely need to focus on communication and education strategies that highlight the importance of penicillin allergies in patient outcomes, in addition to addressing other logistical barriers. The results of this study can inform augmented future delabeling efforts by identifying and addressing specific barriers, improving the clarity of the questionnaire for both nurses and patients, and providing opportunity for follow up allergy assessment for interested patients. Future studies should continue to investigate the optimal methods to implement penicillin delabeling interventions to improve patient outcomes.

## Supplementary material

For supplementary material accompanying this paper visit https://doi.org/10.1017/ash.2022.55. click here to view supplementary material
