# Supplementary material for: Impact of an inpatient nurse-initiated penicillin allergy delabeling questionnaire
Source: Antimicrob Steward Healthc Epidemiol. 2022 May 20;2(1):e86. doi: 10.1017/ash.2022.55 (PMC9726580; doi:10.1017/ash.2022.55)
Supplement: Supplementary file 1 [file S2732494X22000559sup001.docx]

**Supplement Figure 1.** Penicillin allergy de-labeling questionnaire algorithm

**Supplement Figure 2.** Nurse perceptions of patient receptiveness to outpatient allergy evaluation and skin testing
